# Supplementary material for: Crawling and Gliding: A Computational Model for Shape-Driven Cell Migration
Source: PLoS Comput Biol. 2015 Oct 21;11(10):e1004280. doi: 10.1371/journal.pcbi.1004280 (PMC4619082; doi:10.1371/journal.pcbi.1004280)
Supplement: S1 Code — (ZIP) [file pcbi.1004280.s012.zip › release/tst/doc/html/qtgraph_8h_source.html]

Tissue Simulation Toolkit: qtgraph.h Source File


Tissue Simulation Toolkit
 0.1.4.1


- Main Page
- Namespaces
- Classes
- Files

- File List
- File Members

qtgraph.h

Go to the documentation of this file.

1 /\*

2

3 Copyright 1996-2006 Roeland Merks

4

5 This file is part of Tissue Simulation Toolkit.

6

7 Tissue Simulation Toolkit is free software; you can redistribute

8 it and/or modify it under the terms of the GNU General Public

9 License as published by the Free Software Foundation; either

10 version 2 of the License, or (at your option) any later version.

11

12 Tissue Simulation Toolkit is distributed in the hope that it will

13 be useful, but WITHOUT ANY WARRANTY; without even the implied

14 warranty of MERCHANTABILITY or FITNESS FOR A PARTICULAR PURPOSE.

15 See the GNU General Public License for more details.

16

17 You should have received a copy of the GNU General Public License

18 along with Tissue Simulation Toolkit; if not, write to the Free

19 Software Foundation, Inc., 51 Franklin St, Fifth Floor, Boston, MA

20 02110-1301 USA

21

22 \*/

23 #ifndef \_QTGRAPH\_H\_

24 #define \_QTGRAPH\_H\_

25 #include <qwidget.h>

26 #include <qlabel.h>

27 #include <qpainter.h>

28 #include <q3picture.h>

29 #include <qpixmap.h>

30 //Added by qt3to4:

31 #include <QMouseEvent>

32 #include <QPaintEvent>

33 #include <QResizeEvent>

34 #include "graph.h"

35

36

37

38 class QtGraphics : public QWidget, public Graphics {

39

40  Q\_OBJECT

41

42  public:

43  QtGraphics(int xfield, int yfield, const char \*movie\_file=0);

44  QtGraphics(QWidget \*parent, const char \*name, int xfield, int yfield, const char \*movie\_file=0) : QWidget(parent, name) {

45  QtGraphics(xfield, yfield, movie\_file);

46  }

47

48  virtual ~QtGraphics(void);

49  virtual void BeginScene(void);

50  virtual void EndScene(void);

51  /\*inline void Flush(void) {

52  XFlush(display);

53  } \*/

54  virtual void Point( int colour, int i, int j);

55  virtual void Line ( int x1, int y1,int x2,int y2,int colour );

56  /\*void Field (const int \*\*r, int mag=1);

57  void PlotNumber(int number, int x, int y);\*/

58

59  virtual int GetXYCoo(int \*X,int \*Y);

60  /\*char \*ChangeTitle (const char \*message);

61  void RecoverTitle(void);\*/

62  //LineType CropSize(void);

63  //Coordinate ReplaceBeast(Coordinate old\_size,Coordinate new\_size);

64  virtual int XField(void) const {return width();}

65  virtual int YField(void) const {return height();}

66

67  virtual void Write(char \*fname, int quality=-1);

68  inline void ClearImage(void) {

69

70  pixmap->fill(pens[0].color());

71  }

72

73  virtual void TimeStep(void);

74  virtual void resizeEvent( QResizeEvent \*event);

75  public slots:

76  void TimeStepWrap(void);

77

78  signals:

79  void SimulationDone(void);

80

81  private:

82  void paintEvent( QPaintEvent\* );

83  void mousePressEvent( QMouseEvent \*e);

84  void mouseReleaseEvent( QMouseEvent \*e);

85  QPainter \*picture;

86  QLabel \*label;

87  Q3Picture pic;

88  QPen \*pens;

89  QTimer \*timer;

90  QPixmap \*pixmap;

91  QLabel \*image;

92

93  int mouse\_x;

94  int mouse\_y;

95

96  int init\_size\_x;

97  int init\_size\_y;

98

99  double mag;

100  Qt::ButtonState mouse\_button;

101

102  // private methods

103  void ReadColorTable(QPen \*pens);

104  QTimer \*t;

105

106

107

108 };

109

110 #include <qapplication.h>

111 //#include <qsignal.h>

112

113 #define TIMESTEP void QtGraphics::TimeStep(void)

114 #endif

QtGraphics::QtGraphics

QtGraphics(QWidget \*parent, const char \*name, int xfield, int yfield, const char \*movie\_file=0)

**Definition:** qtgraph.h:44

QtGraphics::Line

virtual void Line(int x1, int y1, int x2, int y2, int colour)

Draws a line (obviously... :-)

**Definition:** qt3graph.cpp:107

QtGraphics::XField

virtual int XField(void) const

Returns the width of the Graphics window, in pixels.

**Definition:** qtgraph.h:64

QtGraphics::Point

virtual void Point(int colour, int i, int j)

Plot a point in the Graphics window.

**Definition:** qt3graph.cpp:87

QtGraphics::EndScene

virtual void EndScene(void)

EndScene() must be called to flush the drawing buffer and display the scene.

**Definition:** qt3graph.cpp:100

QtGraphics::BeginScene

virtual void BeginScene(void)

BeginScene() must be called before calling drawing functions.

**Definition:** qt3graph.cpp:94

QtGraphics::Write

virtual void Write(char \*fname, int quality=-1)

Writes the Image to a file.

**Definition:** qt3graph.cpp:200

QtGraphics::QtGraphics

QtGraphics(int xfield, int yfield, const char \*movie\_file=0)

**Definition:** qt3graph.cpp:39

QtGraphics::SimulationDone

void SimulationDone(void)

QtGraphics::TimeStepWrap

void TimeStepWrap(void)

**Definition:** qt3graph.cpp:164

QtGraphics::ClearImage

void ClearImage(void)

**Definition:** qtgraph.h:68

QtGraphics::GetXYCoo

virtual int GetXYCoo(int \*X, int \*Y)

Probes the Window for user interaction, with mouse or keyboard.

**Definition:** qt3graph.cpp:178

graph.h

QtGraphics::~QtGraphics

virtual ~QtGraphics(void)

**Definition:** qt3graph.cpp:81

QtGraphics::TimeStep

virtual void TimeStep(void)

Implement this member function in your simulation code.

Graphics

API for Graphics windows.

**Definition:** graph.h:36

QtGraphics

**Definition:** qtgraph.h:38

QtGraphics::YField

virtual int YField(void) const

Returns the height of the Graphics window, in pixels.

**Definition:** qtgraph.h:65

QtGraphics::resizeEvent

virtual void resizeEvent(QResizeEvent \*event)

**Definition:** qtgraph.cpp:244


---

Generated on Thu Aug 14 2014 22:04:01 for Tissue Simulation Toolkit by  

 1.8.6
